# Single‐Cell Analysis of Preeclamptic Cord Blood Mononuclear Cells Revealed Activation of Heme‐Associated Signalling Pathways

**DOI:** 10.1111/jcmm.71150

**Published:** 2026-04-28

**Authors:** Polina Vishnyakova, Evgeny Karpulevich, Victoria Karyagina, Elena Gantsova, Yulia Trofimovich, Viktoriia Kiseleva, Miroslava Chirkova, Tatiana Gerashchenko, Anastasiya Lokhonina, Kamilla Muminova, Zulfiya Khodzhaeva, Evgeny Denisov, Andrey Elchaninov, Timur Fatkhudinov, Gennady Sukhikh

**Affiliations:** ^1^ National Medical Research Center for Obstetrics, Gynecology and Perinatology Named After Academician V.I. Kulakov of Ministry of Healthcare of Russian Federation Moscow Russia; ^2^ Research Institute of Molecular and Cellular Medicine, Peoples' Friendship University of Russia (RUDN University) Moscow Russia; ^3^ Ivannikov Institute for System Programming of the Russian Academy of Sciences Moscow Russia; ^4^ Avtsyn Research Institute of Human Morphology of Federal State Budgetary Scientific Institution ‘Petrovsky National Research Centre of Surgery’ Moscow Russia; ^5^ Cancer Research Institute, Tomsk National Research Medical Center Tomsk Russia

**Keywords:** heme, PBMC, preeclampsia, single‐cell RNA sequencing, umbilical cord blood

## Abstract

Preeclampsia is a severe gestational complication whose molecular pathogenesis remains poorly understood despite extensive research. It is now recognized that both maternal and fetal cells contribute to disease progression. Notably, single‐cell RNA sequencing of cord blood cells from preeclamptic pregnancies has not been previously investigated, and this became the focus of our study. In this work, we performed flow cytometry, single‐cell RNA sequencing and bioinformatics analysis of cord blood mononuclear cells from preeclampsia and control groups. Bulk RNA‐sequencing data of preeclamptic cord blood from open source was also analysed. We observed a significant decrease in the expression of the CD3, CD8 and CD4 markers in cord blood mononuclear cells in the preeclampsia group. Single‐cell RNA sequencing revealed activation of iron‐ and heme‐associated signalling pathways in various cell types (nonclassical monocytes, naive СD4^+^ and CD8^+^ T cells, T‐helpers 2, NK cells and naive СD4^+^ regulatory T cells) in cord blood during preeclampsia. Analysis of open‐source bulk RNA‐sequencing data confirmed our findings of activation of heme‐related signalling pathways in preeclamptic cord blood samples. These results may suggest the activation of compensatory mechanisms, potentially mediated by the proangiogenic heme degradation products carbon monoxide and biliverdin. Additionally, free heme may act as a proinflammatory damage‐associated molecular pattern in preeclampsia. Further studies are needed to elucidate the direct relationship between these mechanisms and placental heme metabolism.

## 1 Introduction

Preeclampsia (PE) is a pregnancy‐specific syndrome characterized by high blood pressure with the signs of multiple organ damage that usually occurs after 20 weeks of gestation and can lead to serious complications for both mother and fetus. This condition can present with a variety of clinical symptoms, including hypertension, proteinuria, headaches, visual disturbances, swelling and upper abdominal pain. PE can lead to severe complications such as eclampsia, placental abruption and premature birth. PE develops in 8% of all pregnancies, and its aetiology is not completely clear. Researchers have been studying PE for several decades and accumulated a large amount of data on the mechanisms of this serious disease.

Single‐cell RNA sequencing technique is becoming increasingly available and, in the context of PE, has led to major advances in understanding the differences between clinical subtypes of PE and the signalling pathways disrupted in the placenta at PE [1, 2, 3, 4, 5]. Paradoxically, there is still no data in the literature about what changes occur at the level of single‐cell transcriptomics in preeclamptic umbilical cord blood cells. Although attempts to study umbilical cord peripheral blood mononuclear cells (PBMCs) have been made, the amount of data is limited. It was shown that in PE, PBMCs expression of TLR4 was up‐regulated [6] while the levels of T‐helpers 2 and Treg cells were significantly diminished in PE [7]. In the works of Kwon et al. and Gumina et al., a significant decrease in the level of endothelial progenitor cells in cord blood in PE compared to the control group was observed [8, 9]. Hwang and Maeng showed that mesenchymal stem cells (MSCs) obtained from preeclamptic umbilical cord were more senescent, less proliferative, had lower telomerase activity and higher reactive oxygen species production levels than MSCs from a control pregnancy [10]. The work of Pan and colleagues analysed the profile of exosomal microRNA in PE [11]. In general, data on changes in PE umbilical cord blood is limited to the determination of markers of individual cells or analyses such as bulk RNA sequencing or microarray.

The goal of our work was to demonstrate for the first time what happens to the subpopulation cell composition and single cell transcriptomics in umbilical cord blood from pregnant women with PE compared to the control group.

## 2 Material and Methods

### 2.1 Ethics

All experiments with blood were conducted in accordance with the Declaration of Helsinki, guidelines for Good Clinical Practice and Commission of Biomedical Ethics at the Research Center for Obstetrics, Gynaecology and Perinatology, Ministry of Healthcare of the Russian Federation. All experimental protocols were approved by the Commission of Biomedical Ethics at the National Medical Research Center for Obstetrics, Gynaecology and Perinatology of the Ministry of Healthcare of the Russian Federation, Moscow (Ethic's committee approval protocol No. 5, 27 May 2021). All participants signed informed consent in accordance with the Ethics Committee requirements and Helsinki Declaration of the World Medical Association.

### 2.2 Collection of Cord Blood Cells

Umbilical cord blood samples were taken immediately after a planned caesarean section for various clinical indications. The control groups included women who underwent a caesarean section due to the scar after the previous caesarean section, symphysitis and a large size of the fetus. For the PE group, caesarean section was associated with a worsening of the course of PE, fetal growth restriction and the development of HELLP syndrome. Umbilical cord blood was collected using a syringe into a vacuum tube treated with ethylenediaminetetraacetic acid (EDTA) (Sarstedt, Germany). The volume of collected blood was assessed. The blood was diluted with autoMACS Rinsing Solution (Miltenyi biotec, Germany) in a ratio of 1:1. All manipulations with blood in order to level the risk of contamination were performed under aseptic conditions in a laminar flow hood. In addition, umbilical cord blood from patients with PE was subjected to clinical laboratory analysis.

### 2.3 Cord Blood PBMCs Isolation

The isolation of the blood mononuclear fraction was performed using a density gradient of Lympholyte‐H (Cedarlane, Canada) solution as described previously [12]. Briefly, diluted 1:1 with autoMACS Rinsing solution, cord blood was gently layered onto an equal volume of Lympholyte‐H solution and then centrifuged at 400 *g* for 15 min without break at room temperature (RT). A layer of mononuclear cells was carefully collected and washed twice with 300 *g* of autoMACS Rinsing Solution at 11°C for 15 min. Subsequently, the cell pellet was gently resuspended in 900 μL of fetal bovine serum (FBS) (Capricorn Scientific, Germany). The cell count and viability were determined by trypan blue using a Luna‐II cell counter (Logos Biosystems, Republic of Korea). The cells were frozen in the presence of 10% dimethyl sulfoxide (DMSO) (PanEco, Russia) in FBS via slow cooling. Long‐term storage was carried out in liquid nitrogen.

### 2.4 Cell Viability Assessment

Sample preparation for single cell sequencing was performed by thawing cell suspensions in warm water. After thawing, cell suspensions were washed twice in DMEM media (PanEco, Russia) with 10% FBS at 300 *g* 10 min, RT. Supernatant was discarded and the pellet was resuspended in 1 mL of PBS without Ca^2+^ and Mg^2+^ (PanEco, Russia). Cell counting and viability were accomplished with fluorescent dyes acridine orange and propidium iodide (Logos Biosystems, Republic of Korea) on Fluorescence Cell Counter LUNA‐FL (Logos Biosystems, Republic of Korea). Mean viability was 84.35% ± 1.34% for the control group and 79.3% ± 1.84% for the PE group. After counting the samples with highest viability were chosen and cells were diluted with PBS in concentration 1000 cells/μL.

### 2.5 Library Preparation and Sequencing of scRNA

A volume of 8.3 μL of single‐cell suspension, calculated to yield approximately 5000 target cells, was loaded onto the Chromium X platform (10× Genomics, USA) for single‐cell emulsion generation. Single‐cell RNA‐seq libraries were prepared using the Chromium Next GEM Single Cell 3′ Reagent Kits v3.1 (10× Genomics, USA), according to the manufacturer's protocol. Library quality was assessed using the Qsep400 system (BiOptic Inc., China). The peak fragment size ranged from 416 to 448 bp. Libraries were sequenced on the GenoLab M platform (GeneMind, China), using paired‐end sequencing with dual indexing under the following configuration: Read 1: 28 cycles, i7 Index: 10 cycles, i5 Index: 10 cycles, Read 2: 90 cycles.

### 2.6 Processing and QC of scRNAseq Libraries

10× Genomics single‐cell RNA‐seq data were processed using nf‐core/scrnaseq 2.4.1 [13] with Cellranger as an aligner option [14]. Genome assembly GRCh38 was used as a reference. Commands to launch scRNA‐seq data analysis tools are provided in the Supporting Information S1. Intronic reads have been included in the count matrix. SoupX package [15] was used for estimation and removal of cell free mRNA contamination and DoubletFinder—for doublets identification. Imputation was performed using the scImpute method [16].

### 2.7 Clustering and Cell Type Identification of scRNAseq

Imputated data were analysed with Seurat toolkit [17]. We filtered cells that had unique feature counts over 14,000 or less than 500. The SCTransform method was used for normalization and variance stabilization [18]. Batch‐effect correction was performed using Harmony [19]. Cells were labelled with Human Primary Cell Atlas Data, Immune cell expression database and Monaco immune database from celldex [20]. The results were obtained using the equipment of the Shared Research Facility «Shared Research Center of the Ivannikov Institute for System Programming of the Russian Academy of Sciences (SRC ISP RAS).

### 2.8 Differential Expression, Enrichment,Cell–Cell Communication and Open‐source Bulk RNA‐sequencing Data Analysis

Differential expression analysis of single‐cell RNA‐seq data was performed using Seurat. Results were visualized as volcano graphics with Log_2_ Fold Change and −log_10_ adjusted *p*‐value (FDR) axes. A fold change greater than 1.5 or less than −1.5 (0.58/−0.58 as log_2_ Fold Change) was taken as a cutoff for significantly differentially expressed genes. Categorization of genes into molecular pathways based on enrichment analysis was performed in Enrichr (https://maayanlab.cloud/Enrichr/) [21] and Enrichr‐KG programs [22], which also included KEGG database [23]. For cell–cell communication analysis, the CellChat program was used [24]. Bulk RNA‑sequencing data of PE and control cord PBMC samples were taken from open data (GSE154378) [25]. Processing of the open data was carried out using Nextflow nf‐core/rnaseq [26]. Quantitative outputs for individual genes obtained with Salmon from multiple alignments generated using STAR tool, were then used in the Hobotnica pipeline [27] to optimize the choice of tool for differential gene expression analysis. The tool selection algorithm promoted Limma‐Voom tool as the fittest option; the results were filtered by a threshold of 0.1 for adjusted *p*‐values [26]. Quantitative outputs for individual genes obtained with Salmon from multiple alignments generated using STAR tool, were then used in the Hobotnica pipeline [27] to optimize the choice of tool for differential gene expression analysis. The tool selection algorithm promoted Limma‐Voom tool as the fittest option; the results were filtered by a threshold of 0.1 for adjusted *p*‐values.

### 2.9 Flow Cytometry Analysis

Anti‐human monoclonal antibodies from Miltenyi Biotec and Beckman Coulter were incubated with cell suspensions at a density of 0.1 × 10^6^ in 100 μL autoMACS Rinsing Solution (Miltenyi Biotec, Germany) with 0.1% MACS BSA Stock Solution (Miltenyi Biotec, Germany) according to fluorophores compositions presented in Table S1 at 4°C for 10 min in the dark. As a positive control for CD235a staining, whole blood‐EDTA of a healthy donor was diluted 100‐fold in 0.1% MACS BSA Stock Solution and incubated with antibodies according to the same protocol. Following a 500 *g* × 5 min wash with autoMACS Rinsing Solution, cells were resuspended in PBS before analysis was carried out. All samples were analysed on an FACSCalibur flow cytometer (Becton Dickinson, USA) simultaneously after defrosting aliquots of PBMCs of all groups. At least 10,000 cells were collected for each sample. An unstained sample was used as a control and for the mean of fluorescence intensity (MFI) normalization. BD Calibrite 3‐Colour Kit (#340486) and BD Calibrite APC Beads (#340487) were used for the routine calibration of the flow cytometer. Flow cytometry data were either analysed on BD CellQuest (Becton Dickinson, USA) or exported and analysed in FlowJo v10 Software (FlowJo LLC, USA).

The main gating steps are shown in Figure S1. Briefly, logistic gates were made for lymphocytes and monocytes. Monocyte subpopulations were defined by CD14 and CD16 expression levels. CD3 negative and positive populations were determined for lymphocytes. Among CD3 positive cells, cytotoxic T‐lymphocytes and T‐helper cells were identified by CD8 and CD4 distribution, respectively. B‐lymphocyte and NK cell populations were determined by the expression levels of CD19 and HLA‐DR for the former and by CD16 and CD56 expression for the latter among CD3 negative cells.

### 2.10 Statistical Analysis

The data are presented as mean ± standard error mean or as median with interquartile range. The Shapiro–Wilk test was used to estimate the normality of distributions. Data from two groups were compared by Mann–Whitney test or *t*‐test depending on the distribution. All calculations were performed by Prism 7.0 software (GraphPad, USA). *p*‐Value < 0.05 and adjusted *p*‐value (FDR) < 0.05 were considered significant that is, indicative for differences in comparison to the control.

## 3 Results

The main clinical characteristics of patients (*n* = 8) and newborns are shown in Table 1.

**TABLE 1:** Patents' demographic and clinical characteristics.

Characteristics	Control	PE
Number	4	4
Maternal age, years	32.5 ± 2.6	33.97 ± 1.3
Gestational age at delivery, weeks	38.9 ± 0.6	29.75 ± 7.1*
Systolic blood pressure, mm Hg	115.0 ± 3.5	177.0 ± 14.5*
Diastolic blood pressure, mm Hg	72.0 ± 1.6	109.0 ± 14.1*
Proteinuria, g/L	nd	2.6 ± 1.6*
Anaesthesia	Epidural	Epidural/narcosis
Newborn birthweight, g	3392.5 ± 404.9	1908.0 ± 391.4*
Newborn gender (male/female)	3/1	2/2
Intrauterine growth restriction, number of cases	0	1
The total weight of placenta after separation of the umbilical cord and amniotic membranes, g	482.7 ± 104.7	263.0 ± 51.3*

* *p* < 0.05 vs. control group.

It was noted that the values of systolic and diastolic blood pressure, as well as the proteinuria indicator, were significantly higher in groups with PE than in the control group. We also observed significant differences in gestational age, total weight of the placenta after separation of the umbilical cord and amniotic membranes and the newborn weight between the PE and control groups. All newborns from the PE group exhibited clinically significant respiratory disturbances at birth, which required the immediate transfer to the intensive care unit (Table S2). Analysis of cord blood in the PE group revealed a slight decrease in pH and an increase in Base Excess values. Additionally, two cases showed low lactate levels, while one case had elevated lactate.

**FIGURE 1:**
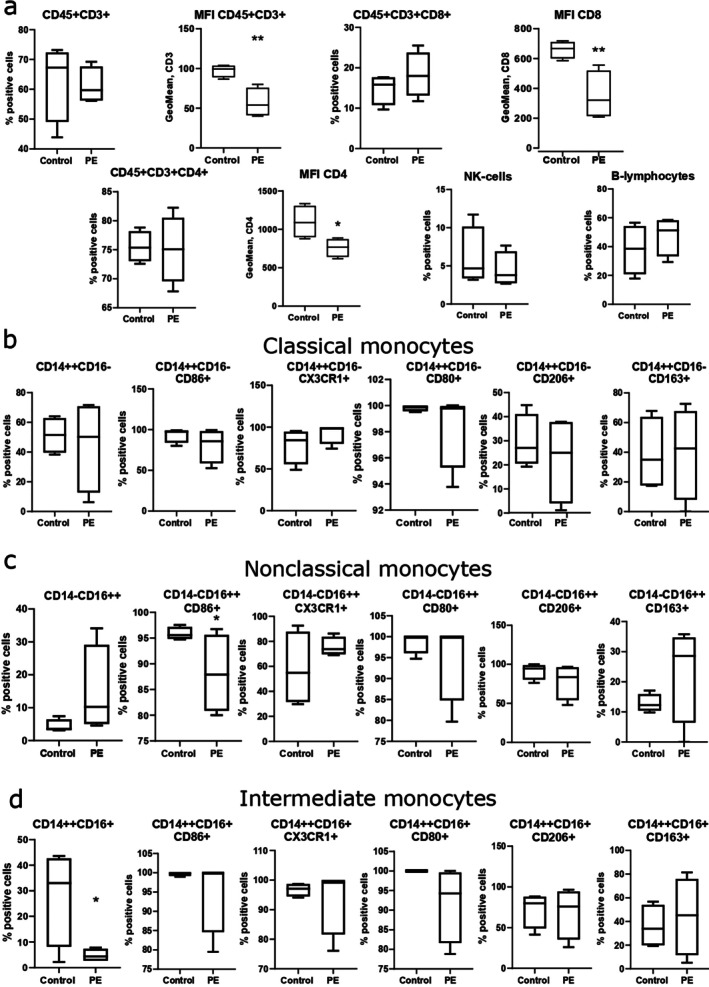
Flow cytometry data. Analysis of cell populations in umbilical cord PBMCs of control and PE groups. (a) T‐lymphocytes (CD45^+^CD3^+^), cytotoxic T‐lymphocytes (CD45^+^CD3^+^CD8^+^), T‐helpers (CD45^+^CD3^+^CD4^+^), with expression of CD4 and CD8 markers on them by estimation of mean of fluorescence intensity (MFI), NK cells, B‐lymphocytes are shown. (b) Assessment of the number of classical CD14^++^CD16^−^ monocytes and the expression of pro‐ (CD86, CX3CR1, CD80) and anti‐inflammatory markers (CD206, CD163) on them after gating. (c) Assessment of the number of nonclassical CD14^−^CD16^++^ monocytes and the expression of pro‐ (CD86, CX3CR1, CD80) and anti‐inflammatory markers (CD206, CD163) on them after gating. (d) Assessment of the number of intermediate CD14^++^CD16^+^ monocytes and the expression of pro‐ (CD86, CX3CR1, CD80) and anti‐inflammatory markers (CD206, CD163) on them after gating. *p < 0.05 by one‐tail *t*‐test, **p < 0.05 by two‐tailed Mann–Whitney test.

Next, we characterized T‐ and B‐lymphocytes, NK cells, monocytes, as well as pro‐ and anti‐inflammatory markers on different monocyte subpopulations of umbilical cord PBMCs using flow cytometry (Figure 1).

Gating strategy for all cell populations is available in Supporting Information (Figure S1). We did not obtain significant differences in the level of cytotoxic T‐lymphocytes (CD45^+^CD3^+^CD8^+^), T‐helpers (CD45^+^CD3^+^CD4^+^), B‐lymphocytes and NK cells in the blood between the groups (Figure 1a). However, in the PE group we obtained a significant decrease in the expression (according to MFI) of the pan‐T cell [28] marker CD3 (*p* = 0.0286), CD8 (*p* = 0.0286) and CD4 (*p* = 0.0336), which are co‐receptor molecules in MHC class I and II‐restricted T cell activation respectively [29] (Figure 1a). Among monocytes (Figure 1b–d), the number of intermediate subpopulation (Figure 1d) was significantly decreased in the PE group (*p* = 0.0251) with no difference in the other monocyte subpopulations.

Then, an in‐depth analysis of monocyte subpopulations was carried out: we assessed classical CD14^++^CD16^−^, nonclassical CD14^−^CD16^++^ and intermediate CD14^++^CD16^+^ monocytes with the expression of pro‐ (CD86, CX3CR1, CD80) and anti‐inflammatory (CD206, CD163) markers on them (Figure 1b–d, Figure S2). A significant difference was found only in CD86^+^ nonclassical monocytes whose number was down‐regulated in the PE group (Figure 1c). A significant increase in the expression level of CX3CR1 marker in classical and nonclassical monocytes was found in the PE group according to the MFI (Figure S2).

**FIGURE 2:**
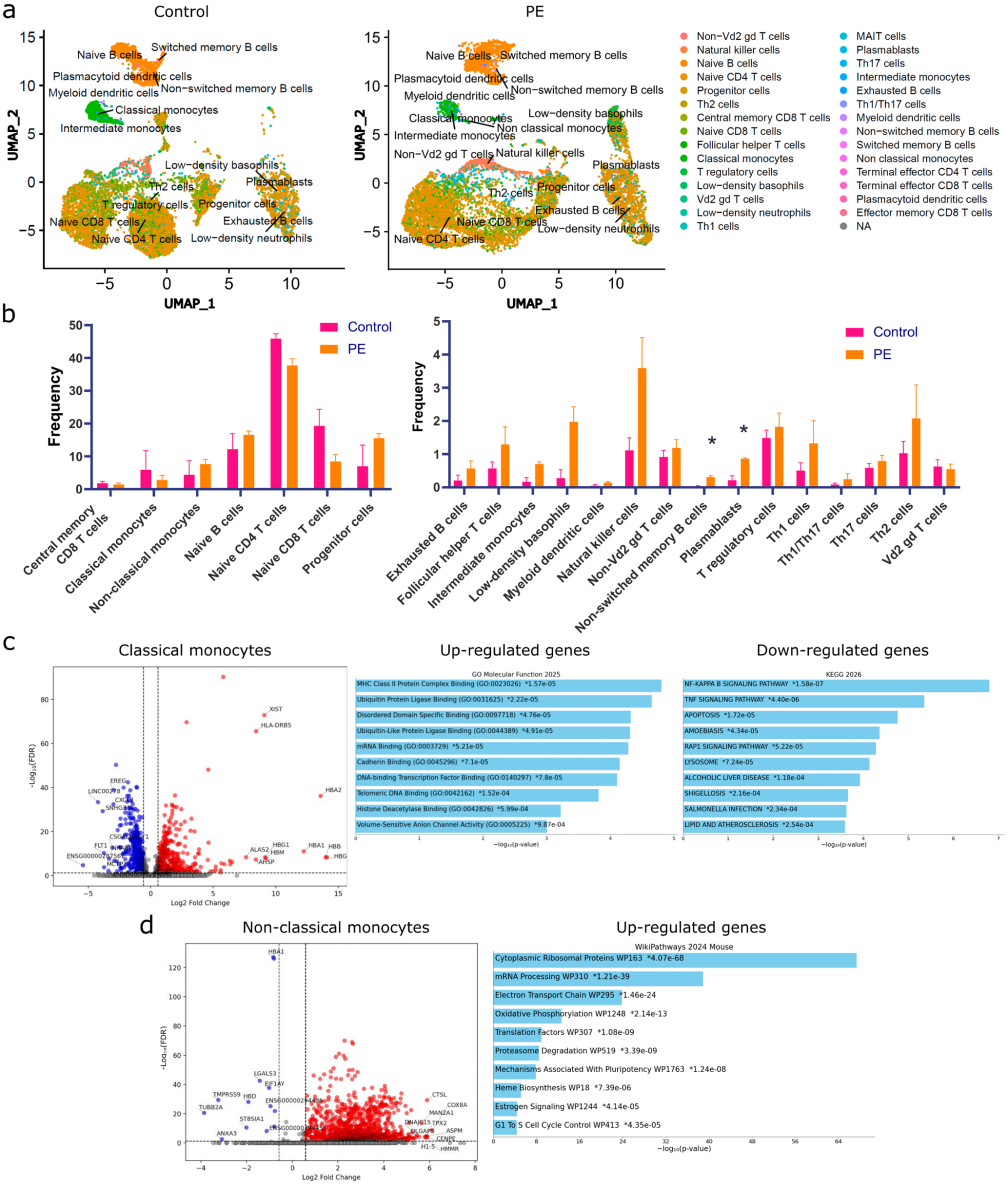
Single cell transcriptomic data of umbilical cord PBMCs in control group and PE. (a) UMAP plot with clusters of PBMCs subsets after clustering, subclustering and cell type annotation in control and PE group. (b) Relative abundance of subsets in control and PE groups, *p < 0.05 vs. control. (c) Volcano plot showing differentially expressed genes of classical monocytes in PE vs. control samples. Red and blue‐coloured genes are statistically significantly differentially expressed (FDR‐adjusted *p*‐value < 0.05 or −log_10_(FDR) > 1.3). The top enriched terms for the significant down‐ and up‐regulated genes set are displayed based on enrichment score and the −log10(*p*‐value), with the actual *p*‐value shown next to each term for classical monocytes. (d) Volcano plot showing differentially expressed genes of nonclassical monocytes in PE vs. control samples with enrichment analysis of up‐regulated genes in nonclassical monocytes. *Next to a *p*‐value indicates the term also has a significant adjusted *p*‐value.

Next, two samples from each group with the maximum viability were subjected to single cell transcriptomic analysis. Quality check parameters are presented in Table S3 and Figure S3. After clustering and annotation with the Monaco immune database, we obtained more than 20 cellular subpopulations as shown in Figure 2a. When analysing the cell frequency, significantly more non‐switched memory B cells and plasmablasts were found in case of PE (Figure 2b).

**FIGURE 3:**
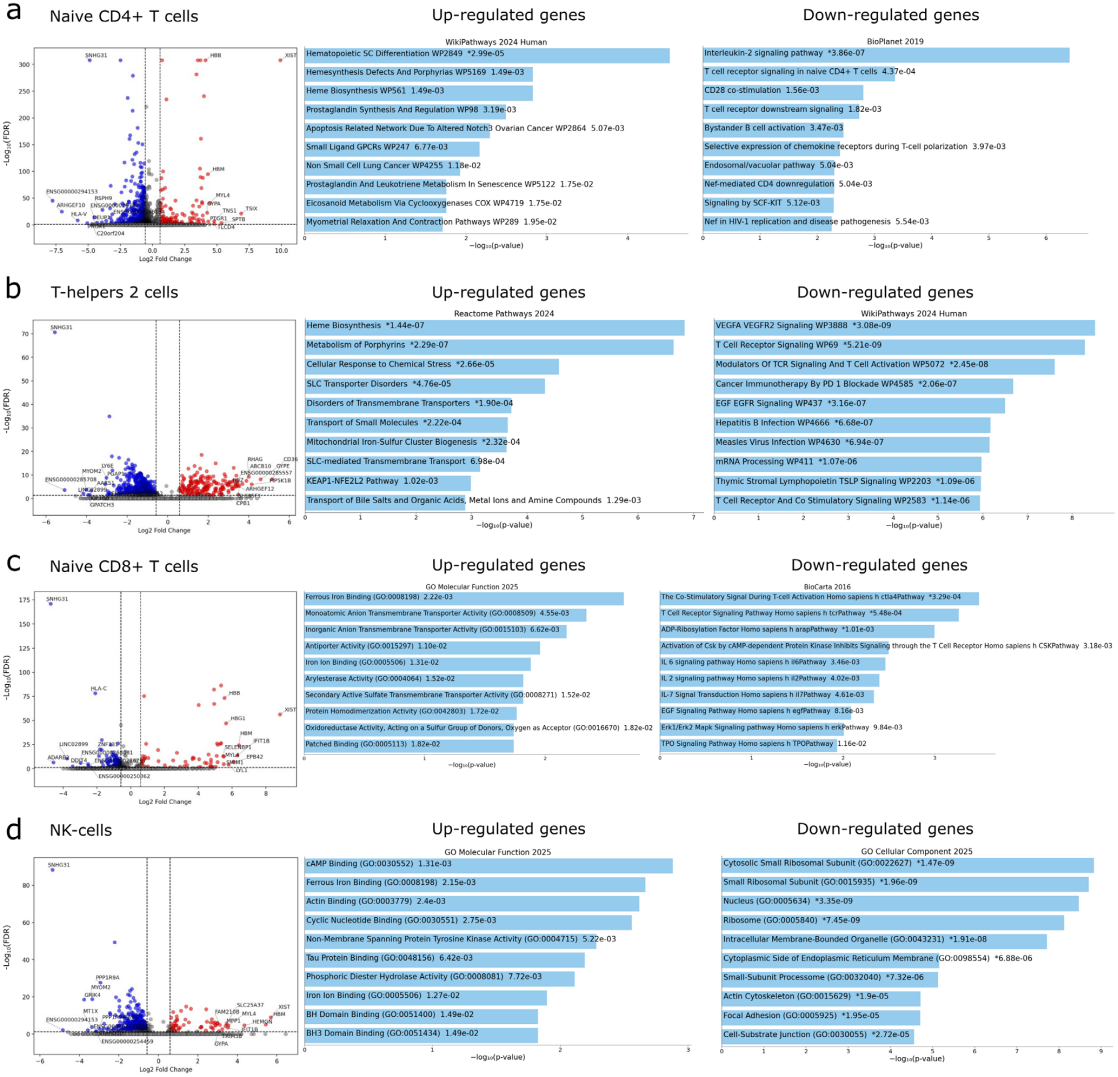
Enrichment analysis of cell subpopulations transcriptomic data in PE vs. control group. (a) Volcano plot showing results of differentially expressed genes of naive CD4^+^ T cells in PE vs. control samples. Red and blue‐coloured genes are statistically significantly differentially expressed (FDR‐adjusted *p*‐value < 0.05 or −log_10_(FDR) > 1.3). The top enriched terms for the significant up‐ (left) and down‐regulated (right) gene set are displayed based on enrichment score and the −log_10_(*p*‐value), with the actual *p*‐value shown next to each term. (b) Volcano plot showing results of differentially expressed genes of T‐helpers 2 cells in PE vs. control samples. Enrichment analysis of down‐ and up‐regulated genes in T‐helpers 2. (c) Volcano plot showing results of differentially expressed genes of naive CD8^+^ T cells in PE vs. control samples. Enrichment analysis of down‐ and up‐regulated genes in naive CD8^+^ T cells. (d) Volcano plot showing results of differentially expressed genes of NK cells in PE vs. control samples. Enrichment analysis of down‐ and up‐regulated genes in NK cells. *Next to a *p*‐value indicates the term also has a significant adjusted *p*‐value.

After clustering, pathway enrichment analysis was performed for significantly up‐regulated and down‐regulated genes separately. For classical monocytes, terms related to MHC class II and ubiquitin protein ligase binding were among the most significant activated signalling pathways, while NF‐Kappa B and TNF signalling pathways were among the suppressed ones (Figure 2c). In nonclassical monocytes, it was possible to conduct an analysis only for the elevated genes (Figure 2d). Among them, terms associated with cytoplasmic ribosomal protein, mRNA processing, oxidative phosphorylation and translation were activated. Another interesting finding was the activated signalling pathway associated with heme synthesis. Later, this pattern was also observed for a number of cells: naive CD4^+^ T cells, T‐helpers 2, naive CD8^+^ T cells, NK cells (Figure 3; left panel) and naive CD4^+^ regulatory T cells (Treg) (Figure S4a,b). Signalling pathways associated with heme synthesis, porphyrin metabolism and iron binding were dominant among the activated genes in these cells.

Regarding the down‐regulated genes, terms associated with IL‐2 signalling, T cell receptor, CD28 costimulation were detected for naive CD4^+^ T cells (Figure 3a right panel). For T‐helpers 2, T cell receptor signalling was also suppressed as well as VEGF and EGF pathways (Figure 3b right panel). Similar patterns were also observed in naive CD8^+^ T cells: T cell receptor signalling, cytokine (IL‐6, IL‐2, IL‐8) and EGF cascades were suppressed (Figure 3c right panel). NK cells (Figure 3d right panel) and CD4^+^ naive Treg (Figure S4c) had suppressed pathways associated with protein synthesis and translation.

In naive B cells, the terms associated with apoptosis and ubiquitination were activated, while the pathways associated with proliferation, transcription and antigen presentation were suppressed (Figure S5).

To examine what happens to cells for which heme synthesis is a primary function, we also annotated the clusters using the Human Primary Cell Atlas (Figure S6a). In the identified erythroblast cluster, there were no heme‐associated signalling pathways among the activated genes; however, there were cascades associated with the cellular response to stress and stimuli (Figure S6b,c).

**FIGURE 4:**
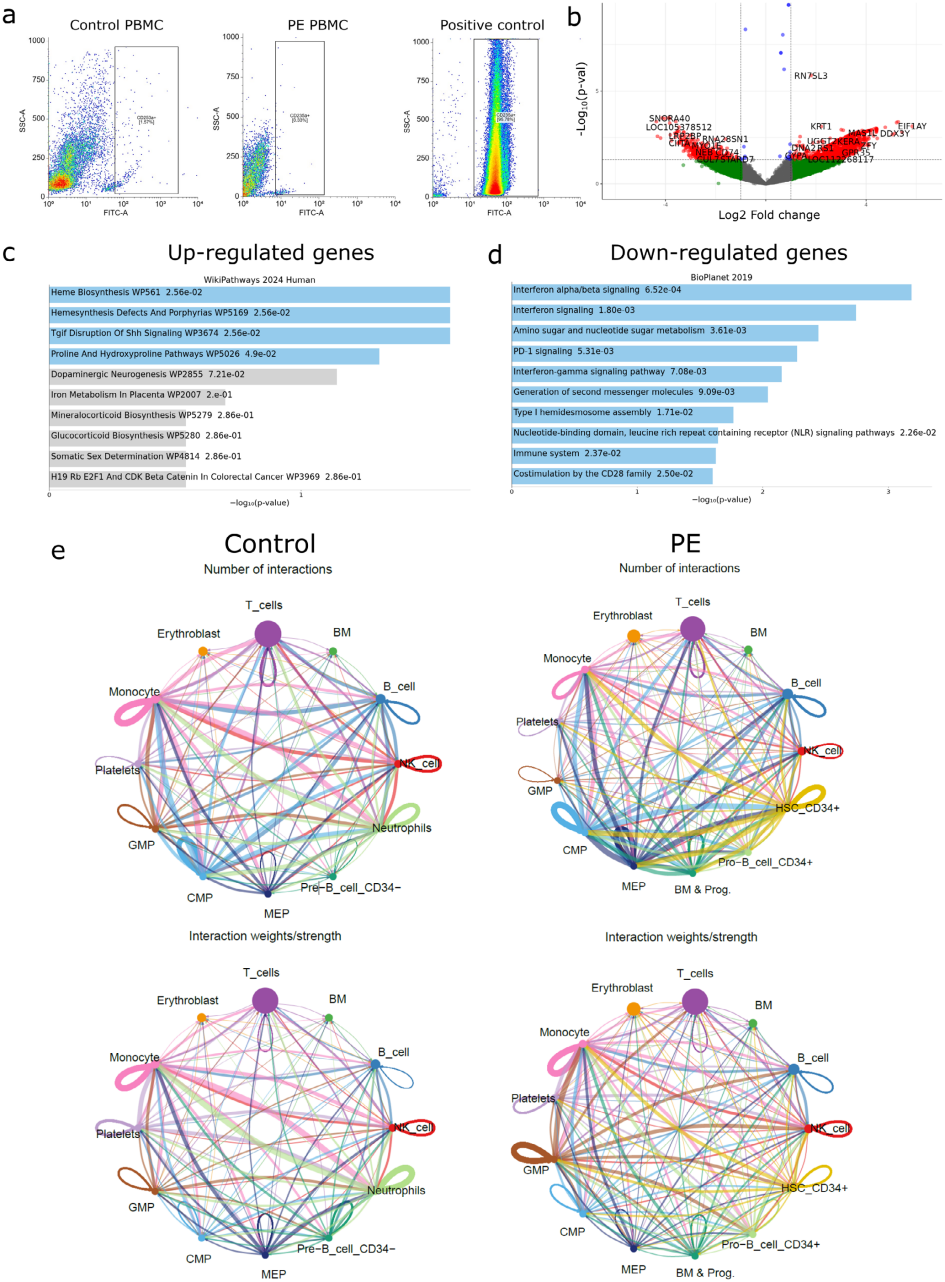
Heme‐associated pathways and cell–cell communications in cord PBMCs in PE vs. control group. (a) Flow cytometry data: representative staining of samples from the control and PE groups for the erythrocyte marker CD235a. Gating was performed on the unstained sample. Diluted whole blood‐EDTA was used as a positive control. (b) Volcano plot showing results of bulk RNA‐seq in PE vs. control PBMCs cord samples from open data (GSE154378) [25]. Red‐coloured genes are statistically significantly differentially expressed (*p* < 0.05 or −log_10_ *p* > 1.3). (c) The top enriched terms for the significant up‐regulated genes of bulk‐RNAseq from open data set are displayed based on enrichment score and the −log_10_(*p*‐value), with the actual *p*‐value shown next to each term. (d) The top enriched terms for the significant down‐regulated genes. (e) Results of significant cell–cell communications in cord blood PBMCs of PE and control group performed in CellChat. Arrow represents a cell–cell interaction, pointing from the sender cell type to the receiver cell type. In the upper panel, thickness is proportional to the number specific interaction. In the lower panel, thickness of the line corresponds to the strength of the predicted interaction. Сircle sizes are proportional to the number of cells in each cell group.

The data obtained regarding heme‐associated signalling pathways inevitably raised the concern of possible sample contamination with erythroid cells. However, filtering during data processing should potentially eliminate erythroid contamination. To verify the presence of erythroid cells, aliquots of the samples that underwent single‐cell sequencing were stained for CD235a, also known as Glycophorin A, a sialoglycoprotein expressed on the membrane of mature erythrocytes and erythroid progenitor cells (Figure 4a). Diluted whole blood‐EDTA was used as a positive control. The presence of CD235a‐positive cells was no more than 3% in both groups (Figure 4a).

To test our hypothesis about activation of heme and porphyrin associated signalling pathways in cord PBMCs during PE, we also used open data of colleagues available in GEO—GSE154378 [25]. We analysed bulk RNA‐seq of all cord blood cells and performed a similar enrichment analysis for significantly up‐ and down‐regulated genes (Figure 4b–d). To our surprise, we found activation of a similar signalling pathway in PE—Heme biosynthesis—among significantly up‐regulated genes (Figure 4c). Among the down‐regulated genes, pathways associated with interferon signalling were predominant.

Then analysis of significantly expressed ligand‐receptor interactions in our single cell data was performed by the CellChat instrument (Figure 4e).

The control group diagram shows a fairly uniform pattern of interactions. The high number of interactions was observed for monocytes, neutrophils, NK cells and Common Myeloid Progenitors (CMPs). In the PE group, there is a higher density of interactions among certain cell types, such as T cells, CMPs and megakaryocyte‐erythroid progenitors, with a prominent interaction with hematopoietic stem cells (HSC) CD34^+^. Next, we analysed such a parameter as weight/strength of interaction, which is expressed in the line thickness and directly corresponds to the strength of the predicted interaction between cell groups. In the control group, this parameter was most pronounced between monocytes and neutrophils and the overall interaction network appeared less dense than in PE. In PE, the most likely interactions were predicted for granulocyte–monocyte progenitors, monocytes and HSC CD34^+^ interacting with other cell types.

## 4 Discussion

Proper interaction of the innate and adaptive immune components influences both the development and course of pregnancy pathologies, including PE. It is important to note that due to the difficulty in obtaining biomaterial for research, PE is most studied from the fetal part of the placenta or by examining changes in peripheral blood of the mother. Our study focused on the molecular signatures of different fetal blood subpopulations and provides the first single‐cell transcriptomics data for cord blood in PE. Among the most important results, the following can be highlighted: we showed that a number of cells (nonclassical monocytes, naive СD4^+^ T cells, T‐helpers 2, naive СD8^+^ T cells, NK cells and naive СD4^+^ Tregs) exhibit enrichment of signalling pathways associated with heme and porphyrin production or iron metabolism among upregulated genes. It is important to note that this result is consistent with open‐access bulk RNA‐seq data of PBMCs in PE. Additionally, in naive СD4^+^, CD8^+^ T cells and T‐helper 2 cells suppression of genes associated with IL‐2, IL‐6, IL‐7 and VEGF/EGF signalling pathways was observed. Ligand‐receptor interactions data revealed the dominant role of progenitor cells and HSC in intercellular communication in PE.

The first part of our work is devoted to flow cytometry analysis of samples taken later in the single‐cell RNA sequencing protocol. One of the most important participants in the proper functioning of adaptive immunity during pregnancy are the T‐lymphocytes. In the PE group, we observed a significant decrease in the expression of CD3, CD8 and CD4 markers [30]. Loewendorf et al., in their work on the study of the phenotype of innate and adaptive immune cells from the cord blood after the birth of 25 children (PE = 9, physiological pregnancy = 16), showed that the cord blood of children born from mothers with PE has a low percentage of CD4^+^ cells, including FoxP3^+^ Treg [31]. Early work by Darmochwal‐Kolarz et al. showed that the blood of newborns after maternal PE showed a decreased percentage of T CD3^+^, CD4^+^ and CD8^+^CD28^+^ lymphocytes [32]. CD4 and CD8 are coreceptors that are physically associated with the T cell receptor (TCR) complex during T cell activation while CD3 is an essential component of TCR [30]. CD4^+^ and CD8^+^ cells have historically been distinguished by the type of antigens they recognize (as part of MHC class II or I molecules, respectively). Thus, it can be assumed that low expression levels of CD4 and CD8 markers on the surface of T cells are closely related to low levels of CD3.

Also, flow cytometry revealed a significant increase in the CX3CR1 expression in classical and nonclassical monocytes in the PE. It is known that the CX3CR1 ligand, CX3CL1, is expressed on endothelial cells and is involved in the underdevelopment of the placental vascular network [33] and inflammation in PE [34]. We previously suggested that the high level of CX3CR1‐positive cells in the decidual membrane of preeclamptic placentas may be due to monocyte colonization of the decidual part of the placenta, associated with endothelial dysfunction and inflammation [35]. Liu and colleagues contributed to the understanding of the involvement of the CX3CL1/CX3CR1 axis in the pathogenesis of PE. CX3CL1/CX3CR1 was shown to be involved in reducing VEGF expression levels in M1 macrophages and leading to impaired HTR‐8/SVneo migration via the FAK signalling pathway [36]. An elevated level of CX3CR1 on monocytes likely indicate inflammation or signs of CX3CL1 expression on the endothelium of the fetus.

One of the most intriguing results was that a number of cells showed increased expression of genes associated with iron‐ or heme‐related signalling pathways. The initial hypothesis upon obtaining these results was sample contamination with erythrocytes or erythroid cells. However, we believe this was not the case, given the rigorous cell filtration performed and low percentage of CD235a positive cells in samples. Additionally, supporting our hypothesis is the absence of similar findings in existing literature on scRNA sequencing of PBMCs or in clusters of erythroblast cells in our data, as well as the concordance of our results with bulk RNA‐seq open data from colleagues [25]. Apparently, in PE, a synchronized pattern of gene expression in several cell populations is observed, aimed at heme synthesis.

Heme, as a prosthetic group, is a component of various proteins and performs functions beyond oxygen transport in red blood cells. Outside of red blood cells, heme serves as a component of enzymes involved in metabolism, detoxification and electron transport in mitochondria [37]. Many enzymes that catalyse oxidation‐reduction reactions contain heme as a cofactor [38]. According to literature data, free heme is considered as a Damage‐Associated Molecular Pattern (DAMP). It activates proinflammatory signalling pathways via TLR4 [39] and the inflammasome [40] and can also contribute to oxidative stress [41]. Heme metabolism is of key importance for placental morphogenesis and pregnancy development. This has been confirmed in both experimental and clinical studies.

Data on heme synthesis in the placenta and the role of this process in the development of specific gestation pathologies is limited. In a rat model, it was found that the gene encoding δ‐aminolevulinate synthase (ALAS‐N), a mitochondrial enzyme that catalyses the rate‐limiting step in heme biosynthesis, is expressed in the trophoblast [42]. Moreover, during gestation, the level of its expression increases. Upon induction of fetal hypoxia, its mRNA level in the placenta of experimental animals rises, indicating that the expression of this gene depends on the level of oxygenation. These data are consistent with our results; that is, activation of heme synthesis pathways indicates hypoxic phenomena often observed in the fetus during PE [43, 44, 45]. Many studies using transcriptome analysis have shown that in PE, the placenta is enriched in signalling pathways associated with iron metabolism [46, 47, 48]. These findings are partially confirmed by Western blot analysis. For example, an increase in ceruloplasmin (a protein synthesized in the liver that plays an important role in iron metabolism and copper transport) level in the placenta has been demonstrated in PE [49]. However, when studying the content of hepcidin and ferroportin, no significant differences were found between PE and normal pregnancies, probably due to higher variability in the levels of these proteins in PE placenta compared to control [50].

At the same time, it is known that activation of enzymes involved in iron transport and accumulation could lead to increased iron concentration in the placenta, which in turn may induce ferroptosis activation in the placenta and contribute to the development of PE [51, 52].

One of the most studied aspects concerning heme and PE is heme oxygenase (HO), which normally breaks down free heme but this process is impaired in PE [53]. Immunohistochemical studies have shown that HO1 is present in trophoblast cells throughout the gestation period [54]. HO1 is known to break down heme into CO, biliverdin and free iron. CO and bilirubin (a product of biliverdin conversion) contribute to the angiogenic, vasodilatory, anti‐inflammatory and antioxidant properties of HO1, thereby counteracting the development of the main signs of PE [55]. Some clinical studies have reported reduced levels of HO1 and CO in the placentas of women with PE [54]. Additionally, in vitro studies have demonstrated that induction of HO1 leads to a decrease in the level of the antiangiogenic factor sFLT‐1. Conversely, in cases of miscarriage during the first trimester, increased expression of the HO1 gene has been observed in the placenta. Considering the cord blood clinical data and respiratory issues of newborns, the potential synthesis of heme by cord PBMCs may be a compensatory response to fetal hypoxia, although there is no direct data indicating that hypoxia occurs. The role of heme‐associated signalling pathway activation in cord blood during PE remains to be elucidated. However, the current results are consistent with heme‐ and iron‐associated alterations occurring in the fetus tissues (including the placenta), according to published data.

The final part of our study investigated potential intercellular interactions in cord blood using single‐cell analysis. Our findings revealed the activation of progenitor and stem cells, along with their enhanced intercellular communication in the PE group. While cord blood is traditionally recognized as a rich source of stem cells, the functional role and effects of their interactions with other blood cell populations remain poorly understood. According to the literature, such interactions can reduce inflammation and carry out immune reprogramming [56, 57]. Interestingly, PE is generally characterized by either a decrease in the number of progenitor and stem cells in cord blood or a reduction in their stemness and differentiation potency [58, 59]. The observed enhancement in intercellular communication may represent a compensatory mechanism to mitigate these deficiencies.

## 5 Conclusion

The present study demonstrates altered signalling pathways associated with heme metabolism in cord PBMCs from preeclamptic pregnancies. These data may indicate an induction of compensatory mechanisms potentially involving the proangiogenic factors CO and biliverdin, which are generated as heme degradation products. It is worth noting, moreover, the role of free heme as a possible proinflammatory DAMP in preeclampsia. Further investigation is required to establish a direct link between these assumptions and placental heme metabolism. Obvious limitations of the study include the small sample size and different gestational ages in the PE and control groups.

## Author Contributions

**Elena Gantsova:** methodology, validation, visualization, writing – review and editing. **Tatiana Gerashchenko:** methodology, validation, investigation. **Evgeny Denisov:** conceptualization, methodology, validation. **Timur Fatkhudinov:** data curation, supervision, resources, project administration. **Polina Vishnyakova:** conceptualization, investigation, funding acquisition, methodology, validation, visualization, writing – original draft, writing – review and editing, software, formal analysis, data curation. **Evgeny Karpulevich:** software, formal analysis, project administration, validation, visualization, writing – review and editing. **Yulia Trofimovich:** software, formal analysis, data curation, visualization, validation, writing – review and editing. **Anastasiya Lokhonina:** investigation, conceptualization. **Miroslava Chirkova:** software, formal analysis, data curation, visualization. **Victoria Karyagina:** methodology, validation, visualization, writing – original draft. **Viktoriia Kiseleva:** methodology, validation, formal analysis, data curation. **Andrey Elchaninov:** conceptualization, investigation, funding acquisition, software, formal analysis. **Kamilla Muminova:** investigation, methodology, validation. **Zulfiya Khodzhaeva:** methodology, validation, investigation, conceptualization. **Gennady Sukhikh:** data curation, supervision, resources, project administration, investigation, funding acquisition.

## Funding

The work was supported by the Ministry of Health state assignment ‘Development of a 3D‐bioresorbable scaffold for breast reconstruction’ no. 1250506058389. Part of the study concerning sequencing analysis was funded by the Russian Science Foundation (RSF) grant number 25‐21‐20111 (https://rscf.ru/project/25‐21‐20111/).

## Ethics Statement

All experiments with blood were conducted in accordance with the Declaration of Helsinki, guidelines for Good Clinical Practice and Commission of Biomedical Ethics at the Research Center for Obstetrics, Gynaecology and Perinatology, Ministry of Healthcare of the Russian Federation. All experimental protocols were approved by the Commission of Biomedical Ethics at the National Medical Research Center for Obstetrics, Gynaecology and Perinatology of the Ministry of Healthcare of Russian Federation, Moscow (Ethics committee approval protocol No. 5, 27 May 2021). All participants signed informed consent in accordance with the Ethics Committee requirements and the Helsinki Declaration of the World Medical Association.

## Conflicts of Interest

The authors declare no conflicts of interest.

## Supporting information


**Table S1:** Fluorophores compositions in flow cytometry.
**Figure S1:** Gating strategy for flow cytometry analysis. In the plot of forward vs. side light scatter, populations corresponding to lymphocytes and monocytes were distinguished by granularity and size. In a plot of pan‐leukocyte marker CD45 expression versus side light scatter, populations corresponding to lymphocytes and monocytes were selected. Logistic gates were created for lymphocytes and monocytes. The subpopulations of monocytes were determined by the expression levels of CD14 and CD16 (right panel of the figure). Pro‐ and anti‐inflammatory markers were then determined within each of the subpopulations.
**Table S2:** Clinical and laboratory characteristics of newborns and cord blood from mothers with PE.
**Figure S2:** Flow cytometry data. Assessment the expression of pro‐ (CD86, CX3CR1, CD80) and anti‐inflammatory markers (CD206, CD163) by mean of fluorescence intensity (MFI) on classical CD14^++^CD16^−^, nonclassical CD14^−^CD16^++^ and intermediate CD14^++^CD16^+^ monocytes after gating. *p < 0.05 by one‐tail *t*‐test.
**Table S3:** MultiQC results for each sample.
**Figure S3:** Characteristics of the samples: eclamps 1, 5: PMBC from PE group; eclamps 3, 6: PMBC from control group.
**Figure S4:** Enrichment analysis of CD4^+^ naïve regulatory T cells (Treg) single cell transcriptomic data in PE vs. control group. (a) Volcano plot showing results PE vs. control samples. Red and blue‐coloured genes are statistically significantly differentially expressed (FDR‐adjusted *p*‐value < 0.05 or −log_10_(FDR) > 1.3). (b) The top enriched terms for the significant up‐regulated genes set are displayed based on enrichment score and the −log_10_(*p*‐value), with the actual *p*‐value shown next to each term. (c) The top enriched terms for the significant down‐regulated genes set are displayed based on enrichment score and the −log_10_(*p*‐value), with the actual *p*‐value shown next to each term. *Next to a *p*‐value indicates the term also has a significant adjusted *p*‐value.
**Figure S5:** Enrichment analysis of naive B cells single cell transcriptomic data in PE vs. control group. (a) Volcano plot showing results PE vs. control samples. Red and blue‐coloured genes are statistically significantly differentially expressed (FDR‐adjusted *p*‐value < 0.05 or −log_10_(FDR) > 1.3). (b) The top enriched terms for the significant up‐regulated genes set are displayed based on enrichment score and the −log_10_(*p*‐value), with the actual *p*‐value shown next to each term. (c) The top enriched terms for the significant down‐regulated genes set are displayed based on enrichment score and the −log_10_(*p*‐value), with the actual *p*‐value shown next to each term. *Next to a *p*‐value indicates the term also has a significant adjusted *p*‐value.
**Figure S6:** (a) UMAP plots with clusters of cord PBMC after clustering, subclustering and cell type annotation in Human Primary Cell Atlas in control and PE group. (b) Volcano plot showing results of differentially expressed genes in erythroblast of PE vs. control samples. Red and blue‐coloured genes are statistically significantly differentially expressed (FDR‐adjusted *p*‐value < 0.05 or −log_10_(FDR) > 1.3). (c) The top enriched terms for the significant up‐regulated genes set are displayed based enrichment score and on the −log_10_(*p*‐value), with the actual *p*‐value shown next to each term.

## Data Availability

The data discussed in this publication have been deposited in NCBI's Gene Expression Omnibus [60] and are accessible through GEO Series accession number GSE266383.

## References

[1] K. A. Campbell , J. A. Colacino , M. Puttabyatappa , et al., “Placental Cell Type Deconvolution Reveals That Cell Proportions Drive Preeclampsia Gene Expression Differences,” Communications Biology 6 (2023): 264, 10.1038/s42003-023-04623-6. 36914823 PMC10011423

[2] I. Admati , N. Skarbianskis , H. Hochgerner , et al., “Two Distinct Molecular Faces of Preeclampsia Revealed by Single‐Cell Transcriptomics,” Medicus 4 (2023): 687–709.e7, 10.1016/j.medj.2023.07.005. 37572658

[3] J. Yang , L. Gong , Q. Liu , et al., “Single‐Cell RNA‐Seq Reveals Developmental Deficiencies in Both the Placentation and the Decidualization in Women With Late‐Onset Preeclampsia,” Frontiers in Immunology 14 (2023): 14, 10.3389/fimmu.2023.1142273. PMC10239844 37283740

[4] E. Knyazev , T. Kulagin , I. Antipenko , and A. Tonevitsky , “scRNA‐Seq of Preeclamptic Trophoblasts Identifies EBI3, COL17A1, miR‐27a‐5p, and miR‐193b‐5p as Hypoxia Markers: Validation of Neuradapt as a Superior Mimetic to Cobalt Chloride,” Placenta 176 (2026): 1–12, 10.1016/j.placenta.2026.02.005. 41666506

[5] X. Wei , K. Li , S. Lu , et al., “Single‐Cell RNA Sequencing Reveals Systemic and Placental Immune Landscape in Preeclampsia,” Placenta 170 (2025): 26–35, 10.1016/j.placenta.2025.08.325. 40803063

[6] G. Xia , D. Xu , M. Wu , and C. Wu , “Expression of Toll‐Like Receptor 4 in Neonatal Cord Blood Mononuclear Cells in Patients With Preeclampsia,” Journal of Huazhong University of Science and Technology. Medical Sciences 30 (2010): 615–619, 10.1007/s11596-010-0552-z. 21063844

[7] M. I. Vargas‐Rojas , H. Solleiro‐Villavicencio , and E. Soto‐Vega , “Th1, Th2, Th17 and Treg Levels in Umbilical Cord Blood in Preeclampsia,” Journal of Maternal‐Fetal and Neonatal Medicine 29 (2016): 1642–1645, 10.3109/14767058.2015.1057811. 26135758

[8] J.‐Y. Kwon , Y.‐S. Maeng , Y.‐G. Kwon , Y.‐H. Kim , M.‐H. Kang , and Y.‐W. Park , “Decreased Endothelial Progenitor Cells in Umbilical Cord Blood in Severe Preeclampsia,” Gynecologic and Obstetric Investigation 64 (2007): 103–108, 10.1159/000100081. 17339774

[9] D. L. Gumina , C. P. Black , V. Balasubramaniam , V. D. Winn , and C. D. Baker , “Umbilical Cord Blood Circulating Progenitor Cells and Endothelial Colony‐Forming Cells Are Decreased in Preeclampsia,” Reproductive Sciences 24 (2017): 1088–1096, 10.1177/1933719116678692. 27879452 PMC6344827

[10] H.‐S. Hwang and Y.‐S. Maeng , “Comparative Analysis of Human Umbilical Cord Blood‐Derived Mesenchymal Stem Cells Between Preeclampsia and Normal Pregnant Women,” Stem Cells International 2020 (2020): 1–15, 10.1155/2020/8403192. PMC7298345 32587622

[11] H.‐T. Pan , X.‐L. Shi , M. Fang , et al., “Profiling of Exosomal microRNAs Expression in Umbilical Cord Blood From Normal and Preeclampsia Patients,” BMC Pregnancy and Childbirth 22 (2022): 124, 10.1186/s12884-022-04449-w. 35152894 PMC8842963

[12] P. Vishnyakova , A. Poltavets , E. Karpulevich , et al., “The Response of Two Polar Monocyte Subsets to Inflammation,” Biomedicine and Pharmacotherapy 139 (2021): 111614, 10.1016/j.biopha.2021.111614. 33930675

[13] F. M. de Almeida , A. Peltzer , G. Sturm , et al., “nf‐core/scrnaseq: 4.0.0,” 2025, 10.5281/ZENODO.15004569.

[14] GitHub—10XGenomics/Cellranger: 10x Genomics Single Cell Analysis accessed July 13, 2025, https://github.com/10XGenomics/cellranger.

[15] M. D. Young and S. Behjati , “SoupX Removes Ambient RNA Contamination From Droplet‐Based Single‐Cell RNA Sequencing Data,” GigaScience 9 (2020): giaa151, 10.1093/gigascience/giaa151. 33367645 PMC7763177

[16] W. V. Li and J. J. Li , “An Accurate and Robust Imputation Method scImpute for Single‐Cell RNA‐Seq Data,” Nature Communications 9 (2018): 997, 10.1038/s41467-018-03405-7. PMC5843666 29520097

[17] Y. Hao , S. Hao , E. Andersen‐Nissen , et al., “Integrated Analysis of Multimodal Single‐Cell Data,” Cell 184 (2021): 3573–3587.e29, 10.1016/j.cell.2021.04.048. 34062119 PMC8238499

[18] S. Choudhary and R. Satija , “Comparison and Evaluation of Statistical Error Models for scRNA‐Seq,” Genome Biology 23 (2022): 27, 10.1186/s13059-021-02584-9. 35042561 PMC8764781

[19] I. Korsunsky , N. Millard , J. Fan , et al., “Fast, Sensitive and Accurate Integration of Single‐Cell Data With Harmony,” Nature Methods 16 (2019): 1289–1296, 10.1038/s41592-019-0619-0. 31740819 PMC6884693

[20] D. Aran , A. P. Looney , L. Liu , et al., “Reference‐Based Analysis of Lung Single‐Cell Sequencing Reveals a Transitional Profibrotic Macrophage,” Nature Immunology 20 (2019): 163–172, 10.1038/s41590-018-0276-y. 30643263 PMC6340744

[21] M. V. Kuleshov , M. R. Jones , A. D. Rouillard , et al., “Enrichr: A Comprehensive Gene Set Enrichment Analysis Web Server 2016 Update,” Nucleic Acids Research 44 (2016): W90–W97, 10.1093/nar/gkw377. 27141961 PMC4987924

[22] J. E. Evangelista , Z. Xie , G. B. Marino , N. Nguyen , D. J. B. Clarke , and A. Ma'ayan , “Enrichr‐KG: Bridging Enrichment Analysis Across Multiple Libraries,” Nucleic Acids Research 51 (2023): W168–W179, 10.1093/nar/gkad393. 37166973 PMC10320098

[23] M. Kanehisa , M. Furumichi , Y. Sato , Y. Matsuura , and M. Ishiguro‐Watanabe , “KEGG: Biological Systems Database as a Model of the Real World,” Nucleic Acids Research 53 (2025): D672–D677, 10.1093/nar/gkae909. 39417505 PMC11701520

[24] S. Jin , C. F. Guerrero‐Juarez , L. Zhang , et al., “Inference and Analysis of Cell‐Cell Communication Using CellChat,” Nature Communications 12 (2021): 1088, 10.1038/s41467-021-21246-9. PMC7889871 33597522

[25] G. Del Vecchio , Q. Li , W. Li , et al., “Cell‐Free DNA Methylation and Transcriptomic Signature Prediction of Pregnancies With Adverse Outcomes,” Epigenetics 16 (2021): 642–661, 10.1080/15592294.2020.1816774. 33045922 PMC8143248

[26] H. Patel , J. Manning , P. Ewels , et al., “nf‐core/rnaseq: nf‐core/rnaseq v3.24.0 – Selenium Seahorse,” 2026, https://zenodo.org/records/19486760.

[27] A. Stupnikov , A. Sizykh , A. Budkina , et al., “Hobotnica: Exploring Molecular Signature Quality,” F1000Res 10 (2021): 1260, 10.12688/f1000research. 36204675 PMC9513410

[28] F. Naeim , P. Nagesh Rao , S. X. Song , and R. T. Phan , “Principles of Immunophenotyping,” in Atlas of Hematopathology (Elsevier, 2018), 29–56, 10.1016/B978-0-12-809843-1.00002-4.

[29] F. Naeim , “Principles of Immunophenotyping,” in Hematopathology (Elsevier, 2008), 27–55, 10.1016/B978-0-12-370607-2.00002-8.

[30] A. M. Mørch , Š. Bálint , A. M. Santos , S. J. Davis , and M. L. Dustin , “Coreceptors and TCR Signaling—The Strong and the Weak of It,” Frontiers in Cell and Developmental Biology 8 (2020): 597627, 10.3389/fcell.2020.597627. 33178706 PMC7596257

[31] A. I. Loewendorf , T. A. Nguyen , M. N. Yesayan , and D. A. Kahn , “Preeclampsia Is Characterized by Fetal NK Cell Activation and a Reduction in Regulatory T Cells,” American Journal of Reproductive Immunology 74 (2015): 258–267, 10.1111/aji.12393. 25962852 PMC5008194

[32] D. Darmochwal‐Kolarz , B. Leszczynska‐Gorzelak , J. Rolinski , and J. Oleszczuk , “Pre‐Eclampsia Affects the Immunophenotype of Neonates,” Immunology Letters 77 (2001): 67–71, 10.1016/S0165-2478(01)00205-X. 11377699

[33] G. Szewczyk , M. Pyzlak , K. Pankiewicz , et al., “The Potential Association Between a New Angiogenic Marker Fractalkine and a Placental Vascularization in Preeclampsia,” Archives of Gynecology and Obstetrics 304 (2021): 365–376, 10.1007/s00404-021-05966-3. 33496844 PMC8277623

[34] M. Siwetz , M. Dieber‐Rotheneder , M. Cervar‐Zivkovic , et al., “Placental Fractalkine Is Up‐Regulated in Severe Early‐Onset Preeclampsia,” American Journal of Pathology 185 (2015): 1334–1343, 10.1016/j.ajpath.2015.01.019. 25769431 PMC4486762

[35] P. Vishnyakova , A. Poltavets , M. Nikitina , et al., “Preeclampsia: Inflammatory Signature of Decidual Cells in Early Manifestation of Disease,” Placenta 104 (2021): 277–283, 10.1016/j.placenta.2021.01.011. 33472135

[36] H. Liu , P. Wang , J. Yin , et al., “High Expression of CX3CL1/CX3CR1 at the Mother‐Fetus Interface of Preeclampsia Inhibits Trophoblast Invasion and Migration,” Placenta 156 (2024): 30–37, 10.1016/j.placenta.2024.08.008. 39236525

[37] A. S. Ogun , N. V. Joy , and M. Valentine , “Biochemistry, Heme Synthesis,” in StatPearls (2023). 30726014

[38] T. L. Poulos , “Heme Enzyme Structure and Function,” Chemical Reviews 114 (2014): 3919–3962, 10.1021/cr400415k. 24400737 PMC3981943

[39] J. D. Belcher , C. Chen , J. Nguyen , et al., “Heme Triggers TLR4 Signaling Leading to Endothelial Cell Activation and Vaso‐Occlusion in Murine Sickle Cell Disease,” Blood 123 (2014): 377–390, 10.1182/blood-2013-04-495887. 24277079 PMC3894494

[40] F. F. Dutra , L. S. Alves , D. Rodrigues , et al., “Hemolysis‐Induced Lethality Involves Inflammasome Activation by Heme,” Proceedings of the National Academy of Sciences 111 (2014): E4110–E4118, 10.1073/pnas.1405023111. PMC4191786 25225402

[41] M. T. Bozza and V. Jeney , “Pro‐Inflammatory Actions of Heme and Other Hemoglobin‐Derived DAMPs,” Frontiers in Immunology 11 (2020): 11, 10.3389/fimmu.2020.01323. 32695110 PMC7339442

[42] N. Ihara , R. Akagi , K. Ejiri , T. Kudo , K. Furuyama , and H. Fujita , “Developmental Changes of Gene Expression in Heme Metabolic Enzymes in Rat Placenta,” FEBS Letters 439 (1998): 163–167, 10.1016/S0014-5793(98)01324-6. 9849899

[43] S. M. Albogami , H. M. Al‐kuraishy , T. J. Al‐Maiahy , et al., “Hypoxia‐Inducible Factor 1 and Preeclampsia: A New Perspective,” Current Hypertension Reports 24 (2022): 687–692, 10.1007/S11906-022-01225-1/METRICS. 36342613

[44] R. Luo , Y. Wang , P. Xu , et al., “Hypoxia‐Inducible miR‐210 Contributes to Preeclampsia via Targeting Thrombospondin Type I Domain Containing 7A,” Scientific Reports 6 (2016): 19588, 10.1038/srep19588. 26796133 PMC4726282

[45] D. S. Charnock‐Jones , “Placental Hypoxia, Endoplasmic Reticulum Stress and Maternal Endothelial Sensitisation by sFLT1 in Pre‐Eclampsia,” Journal of Reproductive Immunology 114 (2016): 81–85, 10.1016/j.jri.2015.07.004. 26228018 PMC4822533

[46] X. Guo , S. Li , and G. Xiong , “Iron Metabolism and Preeclampsia: New Insights From Bioinformatics Analysis,” Journal of Maternal‐Fetal and Neonatal Medicine 38 (2025): 2515416, 10.1080/14767058.2025.2515416;SUBPAGE:STRING:FULL. 40592741

[47] J. Zhong , R. Jiang , N. Liu , et al., “Iron–Immune Crosstalk at the Maternal–Fetal Interface: Emerging Mechanisms in the Pathogenesis of Preeclampsia,” Antioxidants 14 (2025): 890, 10.3390/antiox14070890. 40722994 PMC12292184

[48] N. Yang , Q. Wang , B. Ding , et al., “Expression Profiles and Functions of Ferroptosis‐Related Genes in the Placental Tissue Samples of Early‐ and Late‐Onset Preeclampsia Patients,” BMC Pregnancy and Childbirth 22 (2022): 87, 10.1186/s12884-022-04423-6. 35100981 PMC8805258

[49] S. Guller , C. S. Buhimschi , Y. Y. Ma , et al., “Placental Expression of Ceruloplasmin in Pregnancies Complicated by Severe Preeclampsia,” Laboratory Investigation 88 (2008): 1057–1067, 10.1038/labinvest.2008.74. 18679377 PMC2682720

[50] H. Kim , H. Kim , H. M. Kim , M. J. Kim , H.‐H. Cha , and W. J. Seong , “The Expression of Proteins Associated With Iron Metabolism in the Human Placenta Complicated With Preeclampsia,” American Journal of Obstetrics and Gynecology 228 (2023): S136, 10.1016/j.ajog.2022.11.272.

[51] L. Erlandsson , Z. Masoumi , L. R. Hansson , and S. R. Hansson , “The Roles of Free Iron, Heme, Haemoglobin, and the Scavenger Proteins Haemopexin and Alpha‐1‐Microglobulin in Preeclampsia and Fetal Growth Restriction,” Journal of Internal Medicine 290 (2021): 952–968, 10.1111/JOIM.13349. 34146434

[52] K. E. Gumilar , B. Priangga , C. H. Lu , E. G. Dachlan , and M. Tan , “Iron Metabolism and Ferroptosis: A Pathway for Understanding Preeclampsia,” Biomedicine and Pharmacotherapy 167 (2023): 115565, 10.1016/J.BIOPHA.2023.115565. 37751641

[53] M. L. Zenclussen , N. Linzke , A. Schumacher , et al., “Heme Oxygenase‐1 Is Critically Involved in Placentation, Spiral Artery Remodeling, and Blood Pressure Regulation During Murine Pregnancy,” Frontiers in Pharmacology 5 (2015): 5, 10.3389/fphar.2014.00291. PMC4292788 25628565

[54] R. Inoue , Y. Irie , and R. Akagi , “Role of Heme Oxygenase‐1 in Human Placenta on Iron Supply to Fetus,” Placenta 103 (2021): 53–58, 10.1016/J.PLACENTA.2020.09.065. 33075721

[55] K. Levytska , J. Kingdom , D. Baczyk , and S. Drewlo , “Heme Oxygenase‐1 in Placental Development and Pathology,” Placenta 34 (2013): 291–298, 10.1016/J.PLACENTA.2013.01.004. 23403148

[56] L. Yin , C. Sun , G. Chen , et al., “Modular Mastery of Inflammation: Umbilical Cord Mesenchymal Stem Cells as a Therapeutic Frontier,” Frontiers in Immunology 16 (2025): 1721947, 10.3389/fimmu.2025.1721947. 41488628 PMC12757260

[57] Y. S. Lee , S. K. Sah , J. H. Lee , K.‐W. Seo , K.‐S. Kang , and T.‐Y. Kim , “Human Umbilical Cord Blood‐Derived Mesenchymal Stem Cells Ameliorate Psoriasis‐Like Skin Inflammation in Mice,” Biochemistry and Biophysics Reports 9 (2017): 281–288, 10.1016/j.bbrep.2016.10.002. 28956015 PMC5614481

[58] F. He , W. Cai , S. Cai , et al., “Investigating the Abnormalities and Potential Therapeutic Targets in Umbilical Cord Mesenchymal Stem Cells From Preeclampsia,” Placenta 169 (2025): 49–59, 10.1016/j.placenta.2025.07.077. 40714430

[59] F. Nordin , M. R. M. Idris , Z. A. Mahdy , and S. F. A. Wahid , “Preeclampsia in Pregnancy Affecting the Stemness and Differentiation Potency of Haematopoietic Stem Cell of the Umbilical Cord Blood,” BMC Pregnancy and Childbirth 20 (2020): 399, 10.1186/s12884-020-03084-7. 32650736 PMC7350629

[60] R. Edgar , M. Domrachev , and A. E. Lash , “Gene Expression Omnibus: NCBI Gene Expression and Hybridization Array Data Repository,” Nucleic Acids Research 30 (2002): 207–210, 10.1093/NAR/30.1.207. 11752295 PMC99122

