# Supplementary material for: Single‐Cell Analysis of Preeclamptic Cord Blood Mononuclear Cells Revealed Activation of Heme‐Associated Signalling Pathways
Source: J Cell Mol Med. 2026 Apr 28;30(9):e71150. doi: 10.1111/jcmm.71150 (PMC13125416; doi:10.1111/jcmm.71150)
Supplement: Supplementary file 1 — Table S1: Fluorophores compositions in flow cytometry. Figure S1: Gating strategy for flow cytometry analysis. In the plot of forward vs. side light scatter, populations corresponding to lymphocytes and monocytes were distinguished by granularity and size. In a plot of pan‐leukocyte marker CD45 expression versus side light scatter, populations corresponding to lymphocytes and monocytes were selected. Logistic gates were created for lymphocytes and monocytes. The subpopulations of monocytes were determined by the expression levels of CD14 and CD16 (right panel of the figure). Pro‐ and anti‐inflammatory markers were then determined within each of the subpopulations. Table S2: Clinical and laboratory characteristics of newborns and cord blood from mothers with PE. Figure S2: Flow cytometry data. Assessment the expression of pro‐ (CD86, CX3CR1, CD80) and anti‐inflammatory markers (CD206, CD163) by mean of fluorescence intensity (MFI) on classical CD14++CD16−, nonclassical CD14−CD16++ and intermediate CD14++CD16+ monocytes after gating. *p < 0.05 by one‐tail t‐test. Table S3: MultiQC results for each sample. Figure S3: Characteristics of the samples: eclamps 1, 5: PMBC from PE group; eclamps 3, 6: PMBC from control group. Figure S4: Enrichment analysis of CD4+ naïve regulatory T cells (Treg) single cell transcriptomic data in PE vs. control group. (a) Volcano plot showing results PE vs. control samples. Red and blue‐coloured genes are statistically significantly differentially expressed (FDR‐adjusted p‐value < 0.05 or −log10(FDR) > 1.3). (b) The top enriched terms for the significant up‐regulated genes set are displayed based on enrichment score and the −log10(p‐value), with the actual p‐value shown next to each term. (c) The top enriched terms for the significant down‐regulated genes set are displayed based on enrichment score and the −log10(p‐value), with the actual p‐value shown next to each term. *Next to a p‐value indicates the term also has a significant adjusted p [file JCMM-30-e71150-s001.docx]

**Supplementary Materials**

**Supplementary Materials and methods**

Processing and QC of scRNAseq Libraries. Commands to launch scRNA-seq data analysis tools:

nf-core/scrnaseq 2.7.0 launching command:

nextflow run scrnaseq/main.nf \

> -profile docker \

> --input input.csv \

> --aligner cellranger \

> --outdir results \

> --gtf GRCh38/genome.gtf \

> --fasta GRCh38/genome.fa

versions:

nextflow version 24.10.5.5935

**Table S1. Fluorophores compositions in flow cytometry**

| **Sample** | **Antibody-fluorophore** | **Cat.number** |
| --- | --- | --- |
| 1 | Anti-CD14 FITC | 130-110-518 |
|  | Anti-CD16 PE | 130-113-393 |
|  | Anti-CD45 PerCP-Vio 700 | 130-097-527 |
|  | Anti-CD80 APC | 130-117-719 |
| 2 | Anti-CD14 FITC | 130-110-518 |
|  | Anti-CD16 PE | 130-113-393 |
|  | Anti-CD206 PerCP-Vio 700 | 130-104-129 |
|  | Anti-СX3CR1 APC | 130-096-435 |
| 3 | Anti-CD14 FITC | 130-110-518 |
|  | Anti-CD16 PE | 130-113-393 |
|  | Anti-CD86 PerCP-Vio 700 | 130-116-164 |
|  | Anti-CD163 APC | 130-097-630 |
| 5 | Anti-CD3 FITC | 130-113-138 |
|  | Anti-CD8 PE | 130-125-858 |
|  | Anti-CD4 APC | 130-113-222 |
|  | Anti-CD45 PerCPVio700 | 130-097-527 |
| 6 | Anti-CD16 PE | 130-113-393 |
|  | Anti-CD56 FITC | 130-114-549 |
|  | Anti-CD45 PerCPVio700 | 130-097-527 |
|  | Anti-CD3 АРС | 130-113-697 |
| 7 | Anti-CD3 FITC | 130-113-138 |
|  | Anti-CD19 APC | 130-113-165 |
|  | Anti-CD45 PerCPVio700 | 130-097-527 |
|  | Anti-HLA-DR PE | 130-111-789 |
| 8 | Anti-CD235a FITC | IM2212U |

**
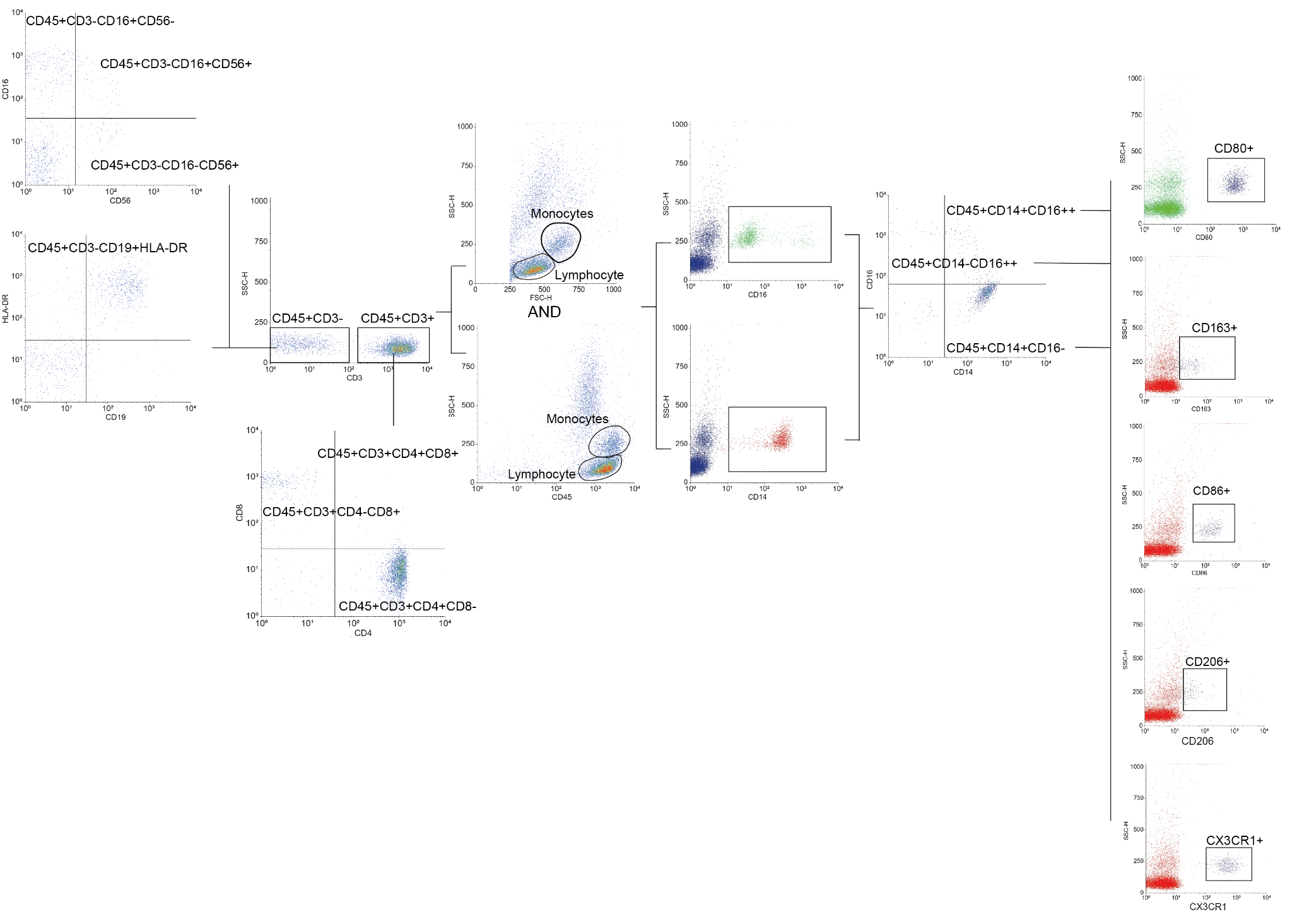
**

Fig.S1. Gating strategy for flow cytometry analysis. In the plot of forward vs. side light scatter, populations corresponding to lymphocytes and monocytes were distinguished by granularity and size. In a plot of pan-leukocyte marker CD45 expression versus side light scatter, populations corresponding to lymphocytes and monocytes were selected. Logistic gates were created for lymphocytes and monocytes. The subpopulations of monocytes were determined by the expression levels of CD14 and CD16 (right panel of the figure). Pro- and anti-inflammatory markers were then determined within each of the subpopulations.

Among the lymphocytes, CD3-negative and CD3-positive subpopulations were identified (left part of the figure). Among the CD3 negative cells, B lymphocytes were identified by the presence of CD19 and HLA-DR, and NK cells by the level of CD14 and CD56 expression. Based on CD4 and CD8 expression, T-killers and T-helpers were identified among CD3-positive cells.

**Supplementary Results**

**Supplementary Table S2.** Clinical and laboratory characteristics of newborns and cord blood from mothers with PE**.**

| Number | 4 |
| --- | --- |
| Сases of artificial ventilation | 4 |
| Fraction of Inspired Oxygen, l/min | 0,266±0,041 |
| Peak Inspiratory Pressure, cmH_2_O | 8,9±0,4 |
| Рositive End-Expiratory Pressure, cmH_2_O | 4,6±0,26 |
| Respiratory rate, per minute | 60,5±1,9 |
| Heart rate, beats/min | 136,75±9,06 |
| pCO_2_, mmHg | 33,5±6,3 |
| SpO_2_, % | 95,25±1,5 |
| pH | 7,31±0,04# |
| Base Excess, mmol/l | 6,3±2,17# |
| Glucose, mmol/l | 2,4±0,2 |
| Lactate, mmol/l | 3,95 ±2,3 |
| Hematocrit, % | 58,73±9,93 |
| Bilirubin, mmol/l | 17,0±2,82 |
| Haemoglobin, g/l | 192,75±26,42 |

# - outside reference values


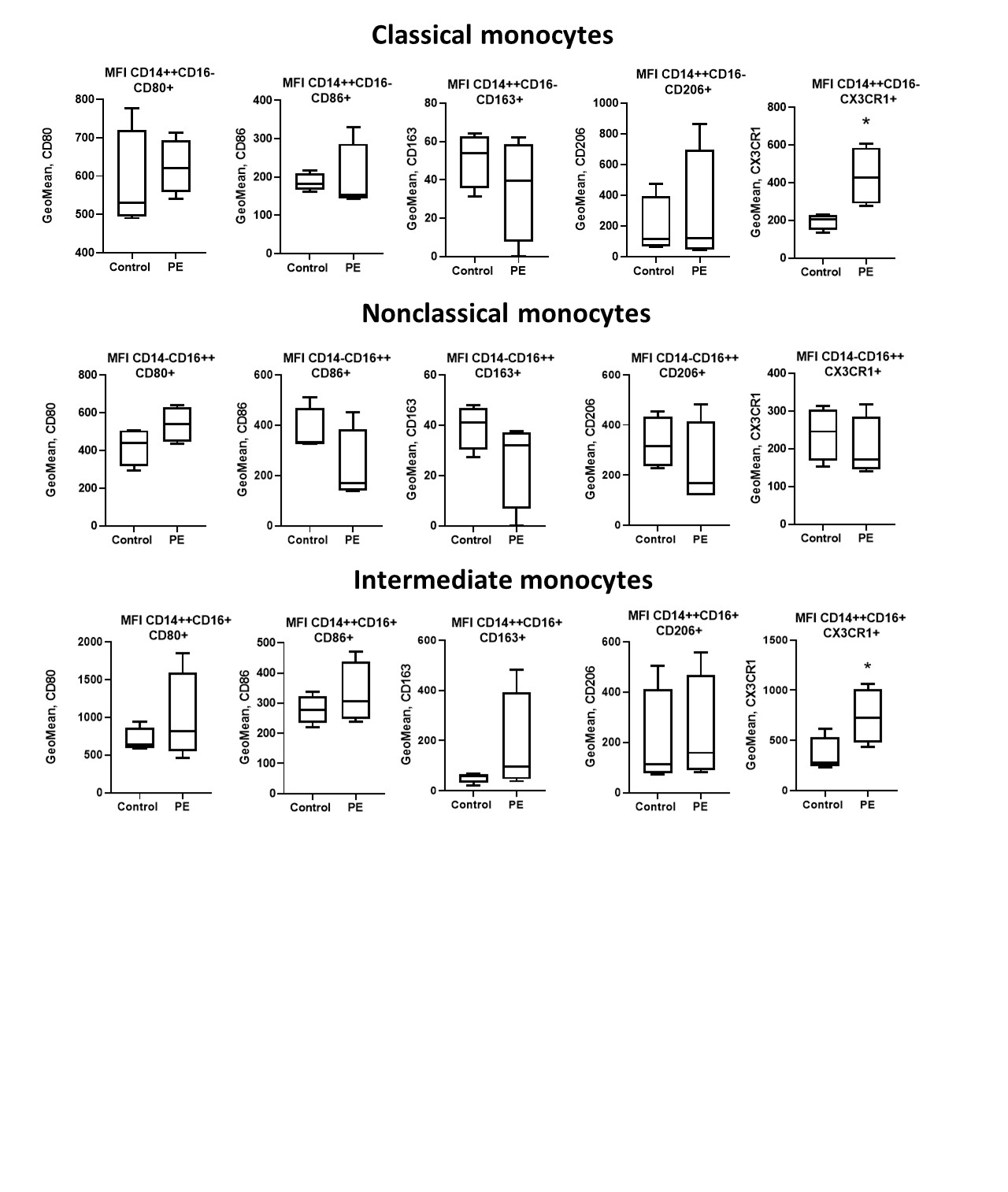


Fig.S2. Flow cytometry data. Assessment the expression of pro- (CD86, CX3CR1, CD80) and anti-inflammatory markers (CD206, CD163) by Mean of fluorescence intensity (MFI) on classical CD14++CD16-, nonclassical CD14-CD16++ and intermediate CD14++CD16+ monocytes after gating. * - p<0.05 by one-tail t-test.

**Supplementary Table S3.** MultiQC results for each sample.

|  |  |  |  |  |
| --- | --- | --- | --- | --- |
|  | **Control 1** | **Control 2** | **PE 1** | **PE 2** |
| Estimated Number of Cells | 5,563 | 5,278 | 13,061 | 4,875 |
| Number of Reads | 91,166,056 | 96,168,325 | 77,642,284 | 72,785,385 |
| Number of Short Reads Skipped | 0 | 0 | 0 | 0 |
| Valid Barcodes | 96.2% | 96.9% | 96.7% | 96.9% |
| Valid UMIs | 99.8% | 99.8% | 99.8% | 99.8% |
| Sequencing Saturation | 28.4% | 33.1% | 15.3% | 26.6% |
| Q30 Bases in Barcode | 97.5% | 97.3% | 97.4% | 97.1% |
| Q30 Bases in RNA Read | 94.0% | 94.8% | 93.9% | 94.0% |
| Q30 Bases in UMI | 95.9% | 95.6% | 95.7% | 95.1% |
| Fraction Reads in Cells | 89.8% | 93.1% | 91.5% | 89.1% |
| Mean Reads per Cell | 16,388 | 18,221 | 5,945 | 14,93 |
| Median UMI Counts per Cell | 4,943 | 6,602 | 1,839 | 4,754 |
| Median Genes per Cell | 1,668 | 2,505 | 211 | 1,771 |
| Total Genes Detected | 34,243 | 37,222 | 33,329 | 34,854 |
| Reads Mapped to Genome | 87.4% | 95.7% | 92.6% | 95.1% |
| Reads Mapped Confidently to Genome | 72.1% | 89.3% | 66.3% | 76.7% |
| Reads Mapped Confidently to Intergenic Regions | 3.1% | 3.9% | 3.2% | 3.8% |
| Reads Mapped Confidently to Intronic Regions | 19.2% | 33.9% | 12.5% | 21.7% |
| Reads Mapped Confidently to Exonic Regions | 49.8% | 51.5% | 50.6% | 51.2% |
| Reads Mapped Confidently to Transcriptome | 54.6% | 71.6% | 48.4% | 58.1% |
| Reads Mapped Antisense to Gene | 9.4% | 11.3% | 6.4% | 9.0% |


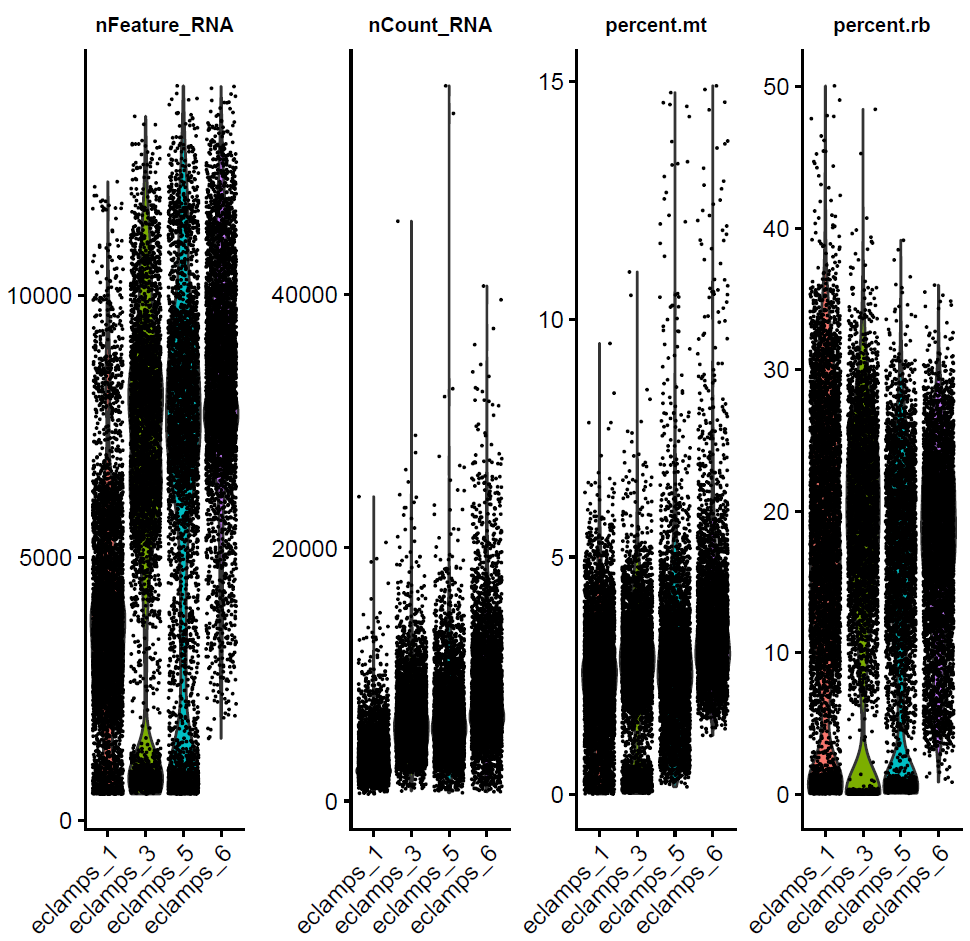


Fig.S3. Characteristics of the samples: eclamps 1, 5 - PMBC from PE group, eclamps 3, 6 - PMBC from control group.


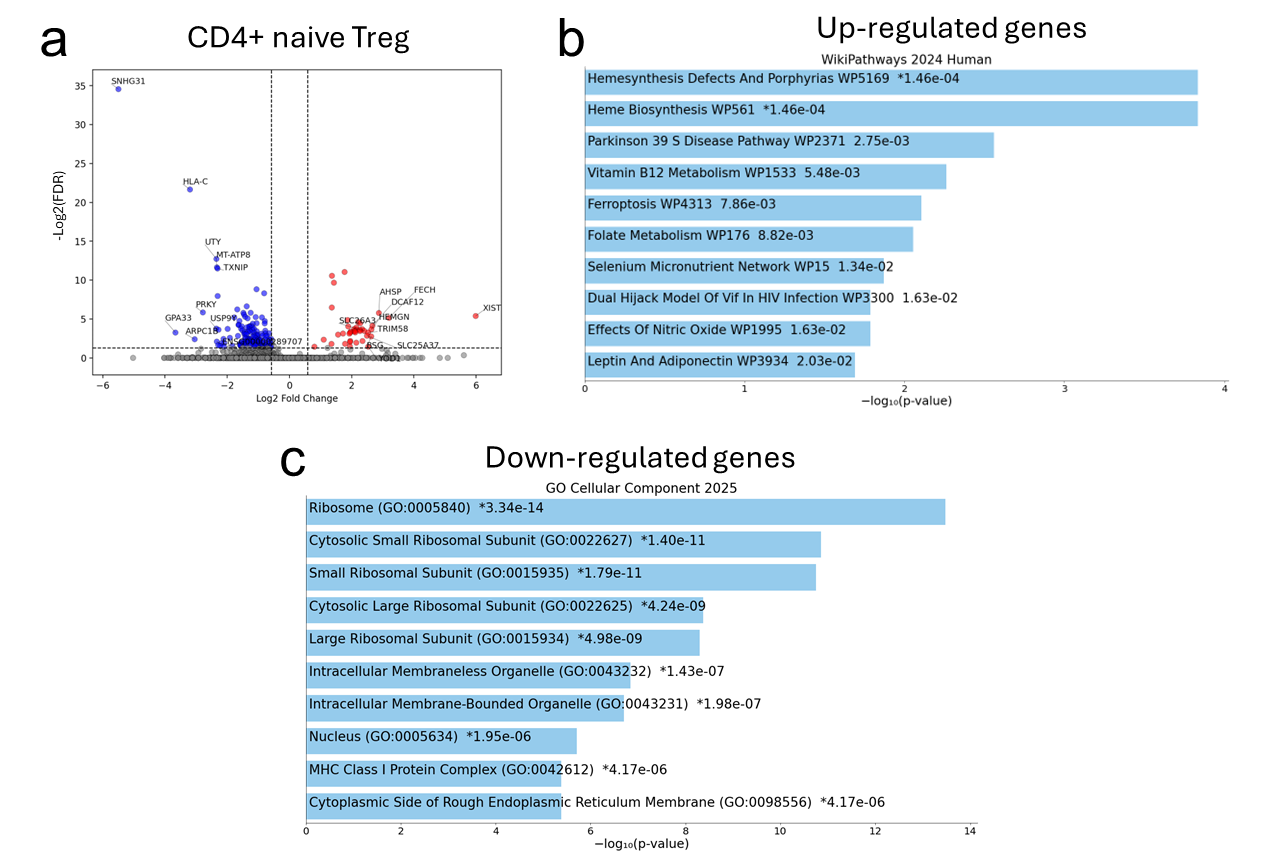


Fig.S4. Enrichment analysis of CD4+ naïve regulatory T cells (Treg) single cell transcriptomic data in PE vs control group. (a) Volcano plot showing results PE vs control samples. Red and blue-colored genes are statistically significantly differentially expressed (FDR-adjusted p-value < 0.05 or -log_10_(FDR) > 1.3). (b) The top enriched terms for the significant up-regulated genes set are displayed based on enrichment score and the -log_10_(p-value), with the actual p-value shown next to each term. (c) The top enriched terms for the significant down-regulated genes set are displayed based on enrichment score and the -log_10_(p-value), with the actual p-value shown next to each term. * next to a p-value indicates the term also has a significant adjusted p-value.


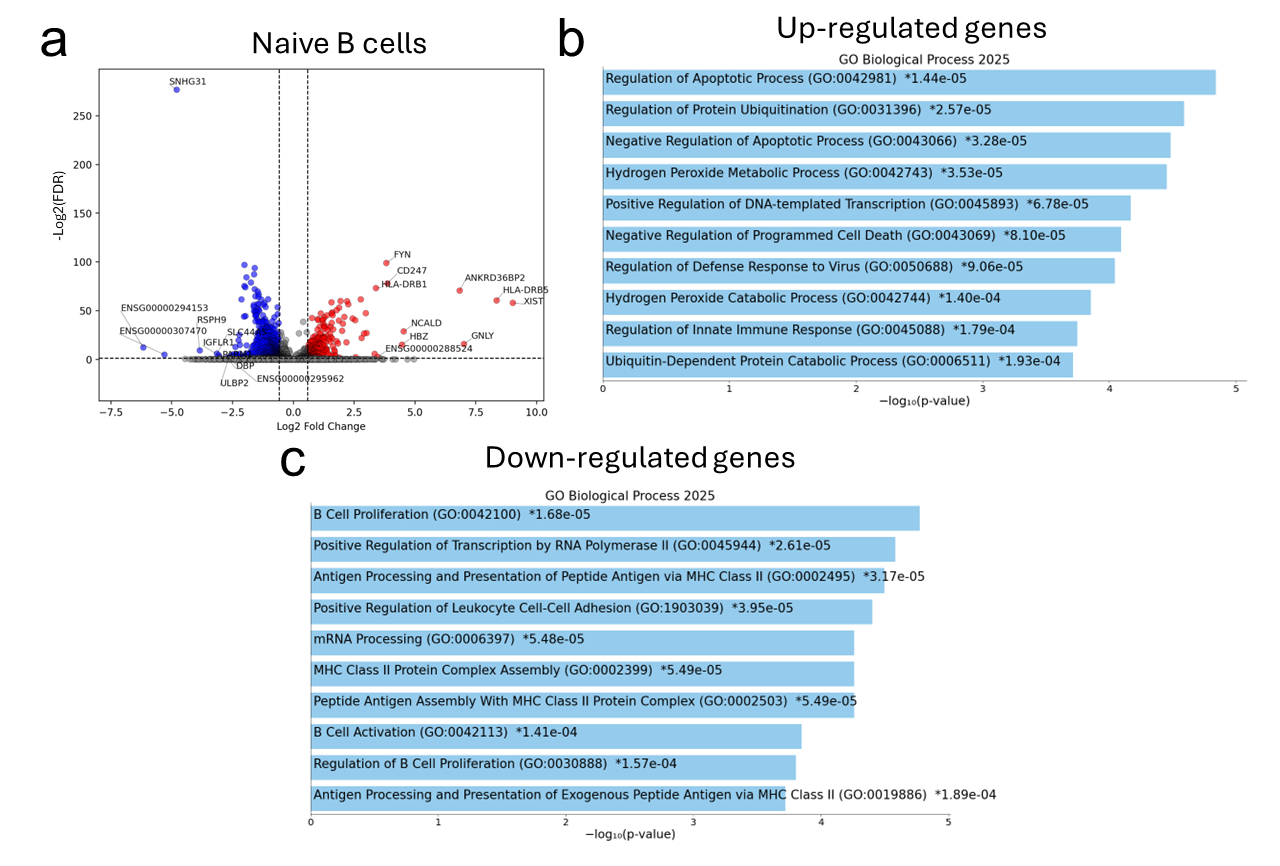


Fig.S5 Enrichment analysis of naive B cells single cell transcriptomic data in PE vs control group. (a) Volcano plot showing results PE vs control samples. Red and blue-colored genes are statistically significantly differentially expressed (FDR-adjusted p-value < 0.05 or -log_10_(FDR) > 1.3). (b) The top enriched terms for the significant up-regulated genes set are displayed based on enrichment score and the -log_10_(p-value), with the actual p-value shown next to each term. (c) The top enriched terms for the significant down-regulated genes set are displayed based on enrichment score and the -log_10_(p-value), with the actual p-value shown next to each term. * next to a p-value indicates the term also has a significant adjusted p-value.


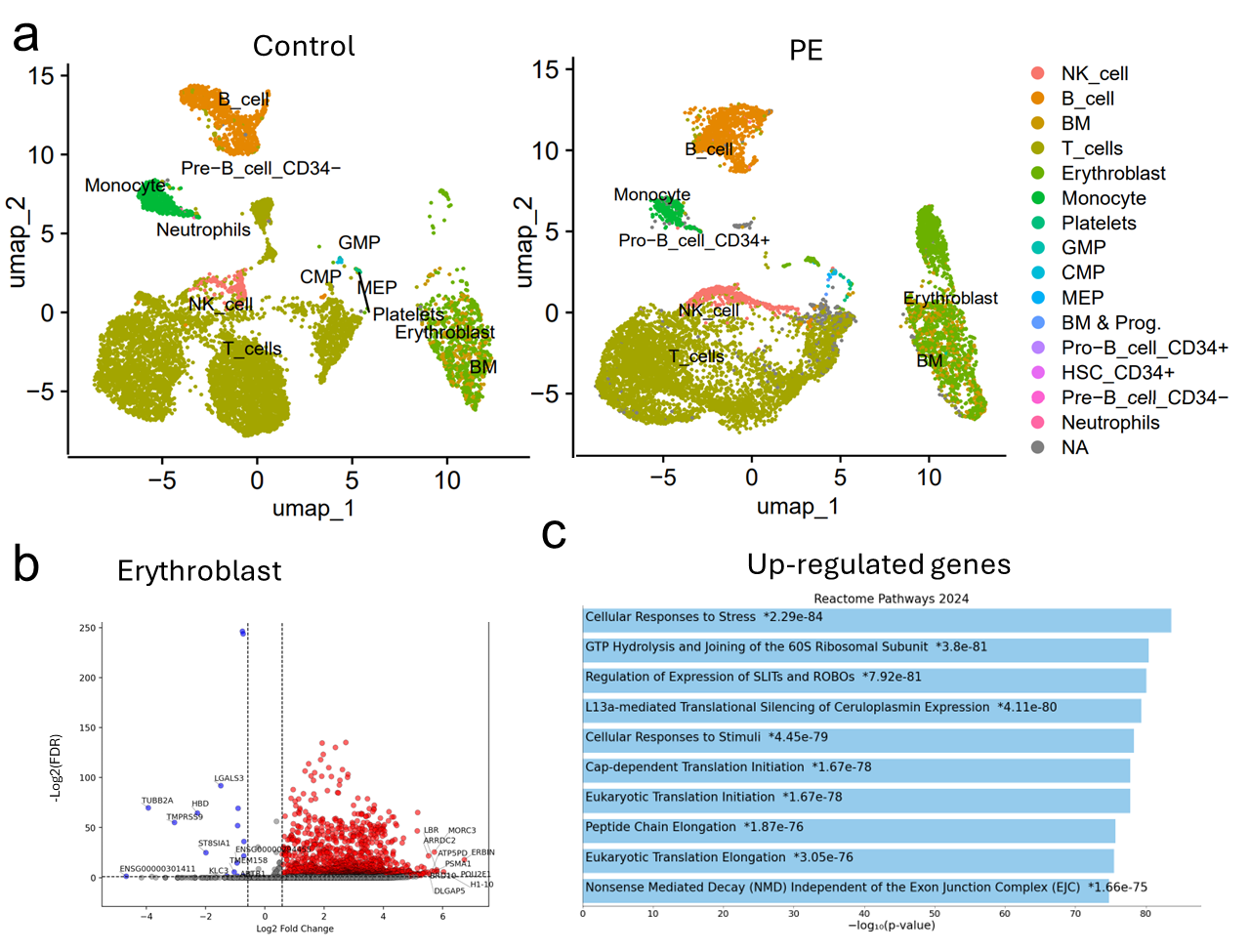


Fig.S6 (a) UMAP plots with clusters of cord PBMC after clustering, subclustering and cell type annotation in Human Primary Cell Atlas in control and PE group.

(b) Volcano plot showing results of differentially expressed genes in erythroblast of PE vs control samples. Red and blue-colored genes are statistically significantly differentially expressed (FDR-adjusted p-value < 0.05 or -log_10_(FDR) > 1.3).

(c) The top enriched terms for the significant up-regulated genes set are displayed based enrichment score and on the -log_10_(p-value), with the actual p-value shown next to each term.

* next to a p-value indicates the term also has a significant adjusted p-value.
